# A bibliometric analysis of studies on gut microbiota in attention-deficit and hyperactivity disorder from 2012 to 2021

**DOI:** 10.3389/fmicb.2023.1055804

**Published:** 2023-03-15

**Authors:** Mingyi Zhao, Yang Meng, Buzi Cao, Jianbin Tong, Xiaoying Liu, Hao Yan, Hanqi Yang, Houzhi Han, Xiaobing Liang, Hui Chen

**Affiliations:** ^1^Department of Pediatrics, The Third Xiangya Hopsital, Central South University, Changsha, Hunan, China; ^2^Medical School, Hunan Normal University, Changsha, China; ^3^Department of Anesthesiology, The Third Xiangya Hopsital, Central South University, Changsha, Hunan, China; ^4^Hunan Province Key Laboratory of Brain Homeostasis, The Third Xiangya Hopsital, Central South University, Changsha, Hunan, China; ^5^Xiangya School of Medicine, Central South University, Changsha, Hunan, China; ^6^Department of Clinical Laboratory, The Third Xiangya Hopsital, Central South University, Changsha, Hunan, China

**Keywords:** gut microbiota, attention-deficit and hyperactivity disorder, bibliometric analysis, visualization analysis, CiteSpace

## Abstract

**Background:**

An increasing number of studies have focused on the role of gut microbiota in the treatment of ADHD, but its related molecular mechanisms are not yet clear, and there is still room for development of studies targeting this area. This study analyzes publications from 2012 to 2021 in a comprehensive and multi-faceted visualization, with the aim of grasping the existing research profile and guiding scholars to make more in-depth studies.

**Methods:**

The 1,677 articles and 298 review articles on gut microbiota in ADHD were retrieved from the Web of Science Core Collection. CiteSpace, VOSviewer, Microsoft Excel 2019, Scimago Graphica, Bibliometrix and Pajek metrics software were used for visualization and analysis of the included literature.

**Results:**

On August 3, 2022, a total of 1975 English-language articles on gut microbiota in ADHD were retrieved from Web of Science Core Collection (WoSCC) from January 2012 to December 2021, with a steady upward trend in the number of articles published in this field over the decade. The top three countries in terms of the number of articles published are the United States, China, and Spain. Meanwhile, CONSEJO SUPERIOR DE INVESTIGACIONES CIENTIFICAS CSIC, UNIV OF CALIFORNIA SYSTEM, and UDICE FRENCH RESEARCH UNIV have made significant contributions in this field. In the analysis of the published journals, *PLoS One* was not only the first in terms of number of articles published but also the most cited. Wang J was the most prolific author and CAPORASO JG ranked first in terms of co-cited authors. In addition, “Diet rapidly and reproducibly alters the human gut microbiome,” published by David LA et al., has the highest citation frequency in this field. The most frequently occurring keyword was “gut microbiota.”

**Conclusion:**

The results of this paper clarify the current status of research on gut microbiota in ADHD. Based on the research on the mechanism of gut microbiota in other diseases, there is reason to believe that the exploration of gut microbiota in ADHD must be increasingly mature. And the study speculates that future research may focus on “nutrition supplements,” “lipid metabolism,” and “gut brain axis.” It is imperative to promote a closer international cooperation among scholars in this field.

## 1. Introduction

The human gastrointestinal tract is the largest micro-ecosystem in the human body, and the large gut microbial community maintains a dynamic balance that sustains the health of the host. It plays an important role in maintaining the health of the organism and in the formation and development of diseases (Thursby and Juge, 2017). The gut microbiota can participate in host metabolism, provide nutrients to the host and stimulate the development of the host immune system (Gomaa, 2020). When the organism suffers from metabolic diseases, cancer, and cardiovascular diseases, it leads to dysbiosis, which means that the ecological balance of the gut microbiota in terms of quality and quantity is altered (Illiano et al., 2020). In turn, gut microbiota dysbiosis is associated with the development of many diseases (Mangiola et al., 2016; Liu et al., 2020; Gong et al., 2021).

Attention-deficit hyperactivity disorder (ADHD) is a neurodevelopmental disorder characterized by persistent disturbances in cognitive functioning patterns that are clinically manifested by symptoms that fall into three categories: hyperactivity, inattention, and hyperimpulsivity (Kalenik et al., 2021), and whose etiology is complex and mainly attributed to susceptibility genes and certain environmental factors. The dopaminergic hypothesis can be used to explain ADHD, whether from animal models, pharmacological perspectives, or brain imaging studies (Genro et al., 2010), and the hypothesis of impaired metabolism of the central catecholamine neurotransmitter norepinephrine has also been implicated in ADHD. Certain gut microbiota not only synthesize neuroactive monoamine molecules (dopamine, norepinephrine, serotonin, and gamma-aminobutyric acid) and their precursors (phenylalanine, tyrosine, tryptophan), but also induce the synthesis of neuroactive compounds in intestinal epithelial cells, and these molecules directly or indirectly have a critical impact on brain function and behavior (Dash et al., 2022). In another way, studies on neuroinflammation have noted the relationship between inflammation and central nervous system development and function, such as inflammatory disease-related genes, and serum inflammatory cytokines (Dunn et al., 2019). In recent years, an increasing number of scholars have become interested in the gut-brain axis. The gut-brain axis is a bidirectional information exchange system that integrates the central nervous system of the brain with the function of the gut (Bull-Larsen and Mohajeri, 2019). The three pathways in the axis include neural pathways, neuroendocrine pathways, and immune pathways (Checa-Ros et al., 2021). Gut microbiota is the starting point of the signaling pathways in the gut-brain axis, so we can develop therapeutic modalities to target the gut-brain axis by regulating the gut microbiota.

Due to the lack of effective treatments, behavioral and psychological therapies are currently mainly used for ADHD (Boonchooduang et al., 2020). Children with ADHD can benefit from alternative therapies such as diet modification, exercise therapy, and nutritional supplements. And natural herbs are also one of the ways to relieve anxiety and depression, which still need further research (Pellow et al., 2011). Although a large number of studies have demonstrated that probiotic supplementation can positively affect the course of neurodevelopmental disorders, long-term and more extensive studies are needed considering the feasibility of their application (Kalenik et al., 2021). In parallel to the above treatments, the emergence of two new therapeutic interventions, fecal microbiota transplantation (FMT), and microbiota transfer therapy (MTT) offers potential benefits for improving ADHD (Xu et al., 2021; Dash et al., 2022). Moreover, due to the complexity of the gut microbiota species and genes, a variety of metabolites are produced, and these metabolites serve as a bridge between the microorganisms and the host, influencing the host physiological and pathological processes (Liu et al., 2022). Therefore, improving microbiota metabolites may also be a target for the treatment of neuropsychiatric disorders. The gut microbiota has a wide range of strain differences, with the in-depth study of gut microbiota, and the accurate identification of microbiota is imminent. Therefore, scholars have invented many analytical tools to analyze the flora, such as DADA (Callahan et al., 2016), Deblur approach (Amir et al., 2017), and QIIME 2system (Bolyen et al., 2019). These tools can achieve accurate and rapid analysis of a variety of microbial characteristics, for the gut microbiota and ADHD research.

### 1.1. Research gaps based on literature review

Based on an extensive literature survey, most of the current studies in the literature focus on three areas: reviewing the potential role of gut microbiota in the pathophysiology of ADHD (Rogers et al., 2016; Eicher and Mohajeri, 2022), analyzing the different possibilities that exist for modulating gut microbiota in the treatment of ADHD (Pelsser et al., 2017; Tang et al., 2022), and comparing differences in gut microbiota between ADHD patients and controls (Prehn-Kristensen et al., 2018; Li et al., 2022; Wang et al., 2022). These are mainly research analyzes of the content of the field, and there are no other quantitative analyzes such as volume of literature, number of authors, number of journals, and countries. Bibliometrics not only detects different topics in the field of study, but also reveals macroscopic patterns in the literature by performing qualitative and quantitative analysis from multiple perspectives based on different data. Therefore, bibliometric analysis of research on gut microbiota in the field of ADHD can help to comprehensively evaluate scientific work and field development.

### 1.2. Research questions and intended contribution of the study

After the above analysis and consideration, this study focused on the following core question:

“Do we need to do a bibliometric analysis of studies on gut microbiota in ADHD?”

The answer is unequivocally yes. It is well known that neuropsychiatric disorders are serious problems that plague every stage of life. ADHD not only has indelible effects on children, but also leads to difficulties in normal life for adults. There is an urgent need to elucidate the molecular mechanisms underlying the pathogenesis of ADHD and to find efficient therapeutic approaches. Groundbreaking research into the role of gut microbiota in brain function and behavior is revolutionizing the way neuropsychiatric disorders have been viewed, offering new hope for the treatment of these disorders. One of the challenges is how to improve ADHD, and improving ADHD through gut microbiota is not a viable treatment option. Obviously, in this context the study of gut microbiota in ADHD in the form of an econometric analysis can provide an in-depth reflection on the field to obtain sufficient valid information and improve the output of the field emphasizing its feasibility to reveal the molecular signaling pathways of disease development. It is meaningful for readers to understand the hot spots in the field in recent years. Consequently, this paper actively explores previous research in this area and provides guidance for possible developments. This paper sets the following objectives for the expected contribution of the study.

### 1.3. Objectives of the present study

With the growth of scientific production, bibliometric studies have become essential to provide relevant information in any field while identifying, highlighting, and viewing scientific knowledge constructed on a topic, theme, or domain of knowledge (Castanha and Grácio, 2014). Gap existing in the research based on the previous research and the expected target are analyzed in metrology:

1. To conduct a comprehensive and multi-faceted visual bibliometric analysis of gut microbiota in ADHD.
2. In order to further clarify the research direction and promote the research progress.

The organization of the rest of the paper is designed as follows: The second section describes the methods and softwares used in the analysis of this paper. Later, Section 3 provides a detailed description of the graphical results for publication numbers, countries, institutions, journals, authors, co-cited literature, keywords, etc. Section 4 discusses the results of this paper in the context of research in the field, and finally, Section 5 and Section 6 summarize the limitations of this paper and the conclusions drawn.

## 2. Methods

### 2.1. Data collection

The Web of Science Core Collection (WoSCC) database is extensively applied in bibliometric analysis, including Science Citation Index Expanded (SCIE) and Social Science Citation Index (SSCI) (Yan et al., 2021; Zhang J. et al., 2021). Data were downloaded from the WoSCC as “Full Record and Cited References” and “Plain Text” on a single day (August 3, 2022), to avoid discrepancies because of daily database updates. The WoSCC was chosen for it is a curated collection of high-quality scholarly peer-reviewed literature published worldwide (Zhu et al., 2020). The search formula was “TS = [(Gut Microbiome OR Gut Microflora OR Gut Microbiota OR Gut Flora OR Gastrointestinal Flora OR Gastrointestinal Microbiome OR Gastrointestinal Microbiota OR Gastrointestinal Microflora OR Gastrointestinal Microbial Community OR Gastrointestinal Microbial Communities OR Gastric Microbiome OR Intestinal flora OR Intestinal Microbiome OR Intestinal Microbiota OR Intestinal Microflora OR Enteric Bacteria OR Gut bacteria) AND (Attention deficit OR ADHD OR ADD)].” The inclusion–exclusion criteria of the literature were as follows:

1. Publications are sourced from 1 January 2012 to 31 December 2021.
2. Only articles and reviews publications were included in this bibliometric study.
3. The publication language was limited to English with no species restrictions.

### 2.2. Data analysis

The WoSCC, CiteSpace 5.8.R3 (Chen, 2006), VOSviewer 1.6.18 (van Eck and Waltman, 2010), Microsoft Excel 2019 and Pajek (Mrvar and Batagelj, 2016), Scimago Graphica, and the R bibliometrix package were used to perform bibliometric analysis and visualization.

From the “Analyze Results” of the WoSCC, we can clearly see annual publications, which were then exported to Excel as data in order to forecast outputs for the next few years. Outputs of authors, countries/regions, and journals were also obtained from it. Moreover, by clicking “View Record” on the treemap visualization map and then entering into the citation report of authors/countries/regions/journals, information about total and annual publications, total citing articles, total times cited, and H-index can be acquired. Furthermore, the “Journal Citation Reports” of the WoSCC exhibits journal impact factor (JIF) and its JIF quartile and category.

CiteSpace is a bibliometric and visual analysis tool that targets at observing cooperation, keywords, internal structure, potential trends, and dynamics in a scientific field (Chen, 2004). Consequently, CiteSpace was used to conduct visualization maps of cooperation between countries, institutions, and authors; analyze co-occurring keywords; detect words with high burstness; and analyze co-cited authors and co-cited journals. Also, we use CiteSpace to carry out reference in-depth mining and cluster analysis, making the information more visible by timeline or timezone view.

VOSviewer is another bibliometric software that is proficient at creating and visualizing knowledge maps and displaying the types of clusters, overlays, and density colors (van Eck and Waltman, 2010; Li et al., 2017). In this bibliometric study, we used VOSviewer to draft keyword co-occurring visualization map.

Microsoft Excel was applied to create a trend chart of annual publications, prognosticate the number of publications in the next few years and conduct a bar chart comparing the number of publications per country annually, a statistical chart of the national publications assessment index (H-index, citations per article, sum of times cited) and tables of the information needed in this article.

Pajek is a powerful tool for studying the existence of various complex nonlinear networks, which runs in the Windows environment and is used for analysis and visualization of large networks with thousands of nodes. In this study, in order to exhibit the categories of important keywords more obviously, we innovatively combined Pajek and VOSviewer for keyword clustering analysis.

Scimago Graphica was applied to construct the visualization map of national cooperation network in the form of and wiring diagram. The R bibliomertix package was used to conduct the analysis of core sources and different collaborative relationships between countries.

## 3. Results

### 3.1. Publication production and forecast 2022 publication production

**Figure 1:**
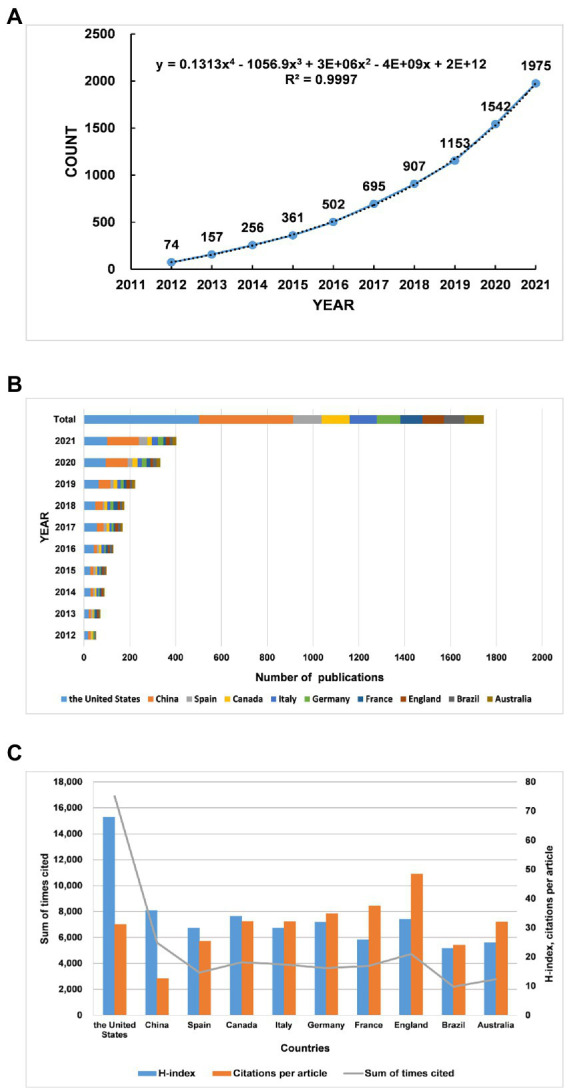
Trends in the number of publications and analysis of country/regions in gut microbiota in attention-deficit and hyperactivity disorder. **(A)** The annual worldwide publication output. **(B)** Growth trends in the publication output from the top 10 countries. **(C)** H-index, citations per article, and total number of citations for the top 10 countries/regions.

By searching the WoSCC for topics related to gut microbiota in ADHD from 2012 to 2021, we could obtain 1975 valid publications that met the nadir criteria, of which 1,677 papers were articles and the remaining 298 were review articles. Figure 1A shows the time distribution of research papers on gut microbiota in ADHD, which shows that the number of articles on gut microbiota in ADHD has been increasing year by year since 2012. In addition, we have examined the number of papers published in each country every year and the number of papers published in 2021 is the highest. In addition, after fitting the annual publication volume of each country, we estimate that the cumulative number of publications in 2022 will be up to 2,402, with a lower confidence interval of 2,270 and an upper confidence interval of 2,534 (Supplementary Table S1; Supplementary Figure S1). The trend of steady increase in the number of publications year by year also proves that this research area is developing and reflects the increasing interest of scholars in this area.

### 3.2. Country/region and institution distribution

**Figure 2:**
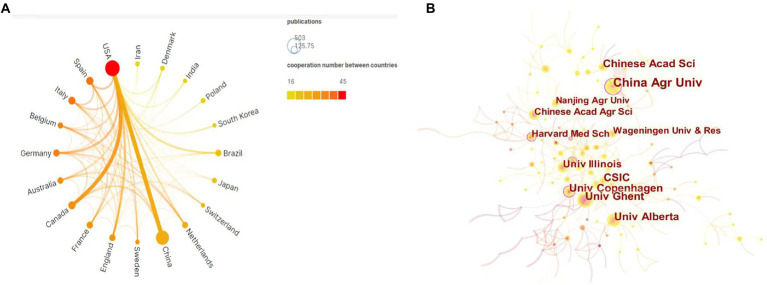
**(A)** Scimago Graphic collaboration visualization map of country/region involved in gut microbiota in attention-deficit and hyperactivity disorder. **(B)** CiteSpace network collaboration visualization map of institutions involved in gut microbiota in attention-deficit and hyperactivity disorder.

The United States is the top country in terms of annual publications for gut microbiota in ADHD from 2012 to 2019, and however, in 2021, China surpasses the United States as the world leader in terms of annual publications (Figure 1B). In the field of gut microbiota in ADHD, the country with the highest number of publications is the United States, accounting for 25% of total publications, followed by China (21%), followed by Spain (125 publications, 6%), Canada (122 publications, 6%), and Italy (119 publications, 6%) (Table 1). Furthermore, we use Scimago Graphica to visualize the collaboration between countries/regions, as shown in Figure 2A. Among them, the node size represents the situation of the amount of articles issued by the country, the larger the node, the more articles issued, the closer the color of the node is to red, and the more the number of articles issued by the country in collaboration with other countries, and the thickness of the connecting line represents the number of collaborations between the two countries. The graph shows that the United States has the closest cooperation with China in this field, followed by Canada.

**Table 1:** Top 10 productive country/regions and institutions.

Rank	Countries/Regions	Articles(*N*)	Percentage(*N*/1975)	H-index	Citations per article	Sum of times cited	Average citation amount	Rank	Institutions	Articles(*N*)	Percentage(*N*/1975)	Location
1	The United States	503	0.25	68	31.20	16,877	33.55	1	CONSEJO SUPERIOR DE INVESTIGACIONES CIENTIFICAS CSIC	43	2.18	China
2	China	409	0.21	36	12.63	5,605	13.70	2	UNIV OF CALIFORNIA SYSTEM	40	2.03	The United States
3	Spain	125	0.06	30	25.47	3,293	26.34	3	UDICE FRENCH RESEARCH UNIV	39	1.97	France
4	Canada	122	0.06	34	32.27	4,095	33.57	4	CHINA AGRICULTURAL UNIV	36	1.82	China
5	Italy	119	0.06	30	32.21	3,915	32.90	5	INRAE	35	1.77	France
6	Germany	102	0.05	32	34.98	3,636	35.65	6	WAGENINGEN UNIV and RESEARCH	32	1.62	Netherland
7	France	97	0.05	26	37.52	3,807	39.25	7	HARVARD UNIV	31	1.57	The United States
8	England	94	0.05	33	48.54	4,726	50.28	8	CHINESE ACADEMY OF SCIENCES	30	1.52	China
9	Brazil	89	0.05	23	24.12	2,207	24.80	9	UNIV OF COPENHAGEN	30	1.52	Denmark
10	Australia	85	0.04	25	32.09	2,774	32.64	10	GHENT UNIV	29	1.47	Belgium

h stands for “high citations,” a scientist has index h if h of his or her Np papers have at least h citations each and the other (Np- h) papers have ≤h citations each(Hirsch, 2005).h index is used to assess not only the scholar’s academic impact but also the country/region’s impact. The United States, with a total number of citations of 16,877 and an h-index of 68, tops the list of countries/regions included. Although China is second only to the USA in terms of total citations (5605) and h-index (36), its citation/article ratio (12.63) is much lower than that of other countries. On the contrary, the citation/article ratios of the England (48.54) and France (37.52) are relatively high, mostly for highly cited papers (Figure 1C; Table 1).

Table 1 lists the top 10 institutions in terms of number of publications, the top three being CONSEJO SUPERIOR DE INVESTIGACIONES CIENTIFICAS CSIC in China (43, 2.18%), UNIV OF CALIFORNIA SYSTEM in the United States (40, 2.03%), and UDICE FRENCH RESEARCH UNIV in France (39, 1.97%). The 10 institutions listed in the table published 345 articles, accounting for 17.47% of the total publications, showing the characteristic of more dispersed research strength. Figure 2B shows the analysis mapping of institutional cooperation made by using CiteSpace. The figure clearly shows that the China Agricultural University institution located in China collaborates with various countries with the most number of collaborations. All of these show that China’s influence in the field is increasing. Additionally, University of Copenhagen, Harvard Medical School, and Wageningen University all have a high degree of intermediary centrality and have importance in the cooperation network.

### 3.3. Journal distribution

Table 2 lists the top 10 most prolific journals and the most co-cited journals in the field of gut microbiota in ADHD. The journals with 50 or more publications were *PLoS One*, *Frontiers in Microbiology*, and *Animals*, where *PLoS One* not only ranked first in the top 10 most prolific journals with 58 publications (2.94%), but also had the highest h-index (26). 2021, *Gut Microbes* was the top 10 high-yielding journals with the highest IF (9.434), followed by *Food Research International* (7.425), and *Nutrients* (6.706). Overall, the number of journals with high impact factors is small, and there is still much room for improvement. Among the top 10 high-producing journals, only two journals, *PLoS One* and *Scientific Reports*, belong to Q2 (25–50%), and the remaining eight journals belong to Q1 region (the top 25% of the IF distribution).

**Table 2:** Top 10 productive journals and co-cited journals.

Rank	Journal	Count(N)	Percentage(N/1975)	IF (2021)	H-index	Quartile in category	Rank	Co-cited Journal	Count(N)	IF (2021)	H-index	Quartile in category
1	PLoS One	58	2.94	3.752	26	Q2^b^	1	PLoS One	1,085	3.752	26	Q2^b^
2	Frontiers in Microbiology	55	2.78	6.064	17	Q1^c^	2	Applied and Environmental Microbiology	853	5.005	12	Q2^c^
3	Animals	54	2.73	3.231	11	Q1^a^	3	Nature	799	69.504	90	Q1^b^
4	Nutrients	40	2.03	6.706	14	Q1^d^	4	Proceedings of the National Academy of Sciences of the United States of AME	789	10.700	68	Q1^b^
5	Scientific Reports	36	1.82	4.996	19	Q2^b^	5	Science	642	63.798	76	Q1^b^
6	Poultry Science	33	1.67	4.014	17	Q1^a^	6	Scientific Reports	559	4.996	19	Q2^b^
7	Journal of Animal Science	28	1.14	3.338	13	Q1^a^	7	Journal of Nutrition	550	4.687	39	Q2^d^
8	Aquaculture	27	1.37	5.135	12	Q1^e^	8	Frontiers in Microbiology	534	6.064	17	Q1^c^
9	Food Research International	24	1.22	7.425	13	Q1^f^	9	British Journal of Nutrition	529	4.125	34	Q3^d^
10	Gut Microbes	23	1.16	9.434	14	Q1^g^	10	Gut	504	31.840	112	Q1^g^

^a^Agriculture, Dairy and Animal Science. ^b^Multidisciplinary Sciences. ^c^Microbiology. ^d^Nutrition and Dietetics. ^e^Fisheries. ^f^Food Science and Technology. ^g^Gastroenterology and Hepatology. ^h^Biochemistry and Molecular Biology.

Notably, *PLoS One* not only has the highest number of publications in the field, but also the highest number of citations (1,085 citations). *PLoS One* has played a significant role in advancing research on gut microbiota in ADHD. Among the top 10 most cited journals, *Nature* and *Science* have the highest impact factor, with IFs of 69.504 and 63.798 in 2021, respectively. In addition, the journal with the highest h-index (112) is *Gut*, followed by *Nature* (90) and *Science* (76). Among the top 10 most cited journals, half of the journals were in Q1, four were in Q2, and only one was in Q3.

**Figure 3:**
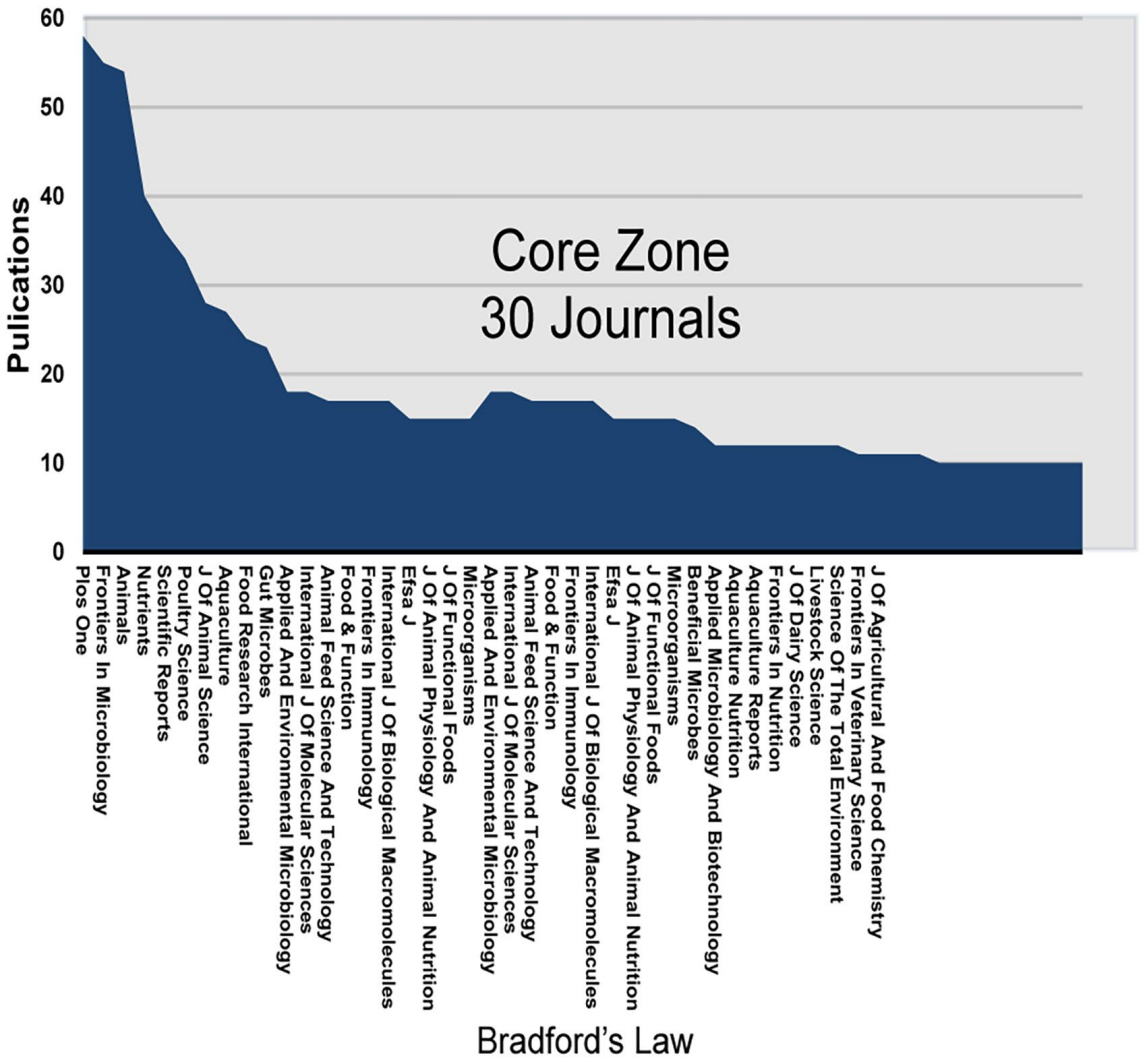
Source clustering through Bradford’s Law.

Bradford’s law is often used to verify patterns in the distribution of literature in journals. Bradford’s law is one of the most important laws of bibliometrics, and is described as a decreasing order of scientific journals by the number of papers they contain on a subject, so that among all these journals, the core section with the highest rate of publication and the subsequent sections containing the same number of papers as the core section can be distinguished. The number of magazines contained in the core and subsequent zones is 1:α:α^2^:⋯ (α>1; Bradford, 1934). Figure 3 shows the partitioning of journals in this field based on Bradford’s law using bibliometrix, where all journals are divided into three zones and the number of papers in each zone is approximately the same. The ratio of the number of journals is calculated to be close to 1:4:16. (1,4:4^2^) (Supplementary Table S2).

### 3.4. Author distribution

**Figure 4:**
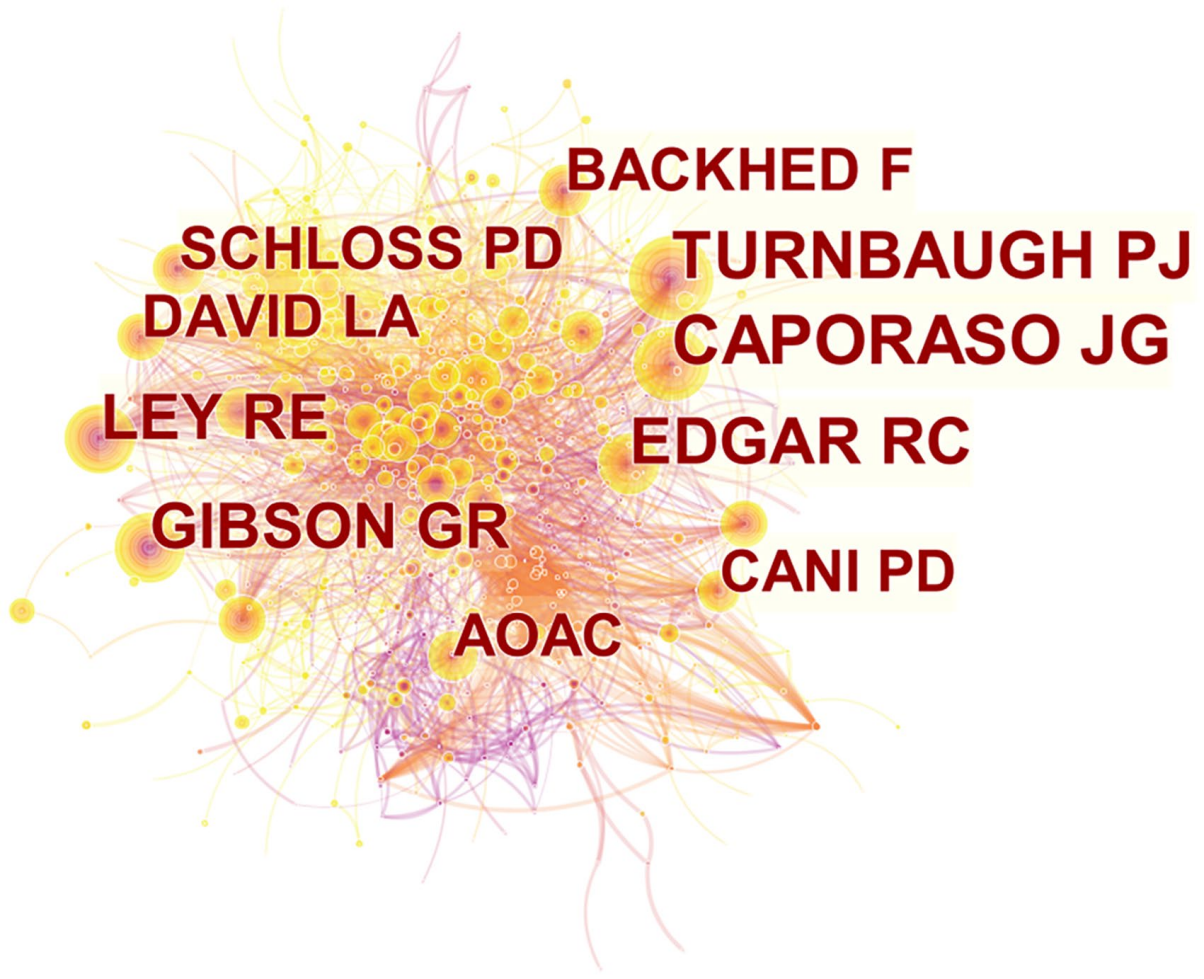
CiteSpace network visualization map of co-cited authors of the articles related to gut microbiota in attention-deficit and hyperactivity disorder.

Research cannot develop and progress without the hard work of a large number of research scholars. We can clearly see that the top 10 most productive authors in the field of gut microbiota in ADHD have little difference in the number of published articles, the first Wang J has 15 articles from China and the tenth Crebelli R has 12 articles from Italy (Table 3). Figure 4 shows the network map of co-cited authors generated by CiteSpace. The node size is proportional to the number of citations. Among the top 10 co-cited authors, CAPORASO JG is the most highly co-cited author (217 citations), followed by TURNBAUGH PJ (168 citations) and EDGAR RC (161 citations). These authors have a strong presence in the field and the quality of their articles is high. The closer the authors are connected to each other, the more similar their research topics are.

**Table 3:** Top 10 productive authors and co-cited authors.

Rank	Author	Count	Rank	Co-cited author	Citation
1	Wang J	15	1	CAPORASO JG	217
2	Dusemund B	13	2	TURNBAUGH PJ	168
3	Gundert-remy U	13	3	EDGAR RC	161
4	Lambre C	13	4	GIBSON GR	147
5	Li Y	13	5	LEY RE	138
6	Mortensen A	13	6	AOAC	99
7	Waalkens-berendsen I	13	7	BACKHED F	96
8	Wang Y	13	8	DAVID LA	96
9	Aguilar F	12	9	SCHLOSS PD	91
10	Crebelli R	12	10	CANI PD	90

**Figure 5:**
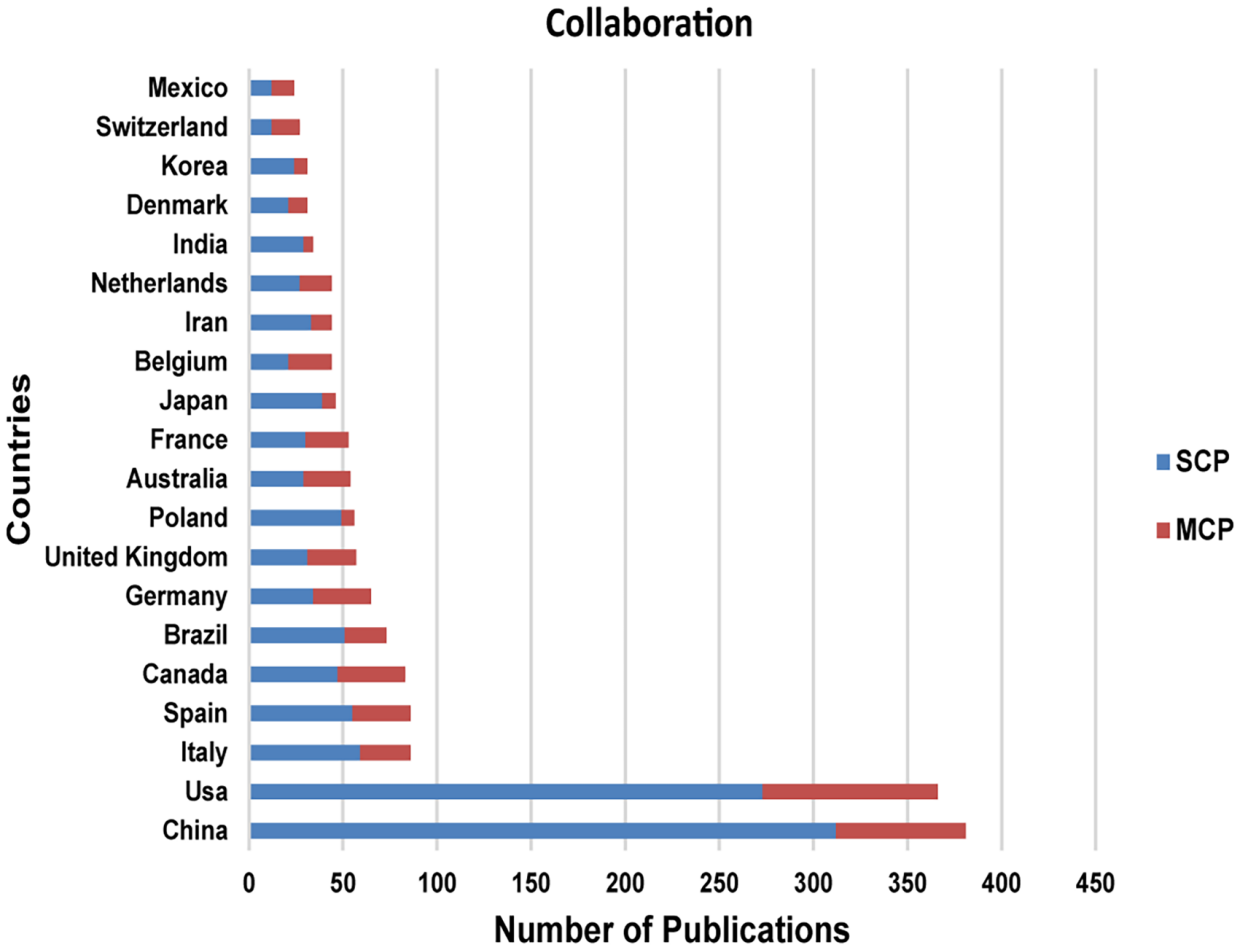
Corresponding Author’s Country.

Besides, we also analyze the nationality of corresponding author, where MCP indicates the number of co-authored papers with authors from other countries and SCP indicates the number of co-authored papers with authors from the same country (Figure 5). As is shown in the chart, the number of co-authored papers in China and the United States far exceeds that in other countries. What’s more, compared with the United States, China not only has a higher total number of co-authored papers, but also has a majority of co-authored papers of the same country.

### 3.5. Co-cited references and co-cited clusters analysis

**Figure 6:**
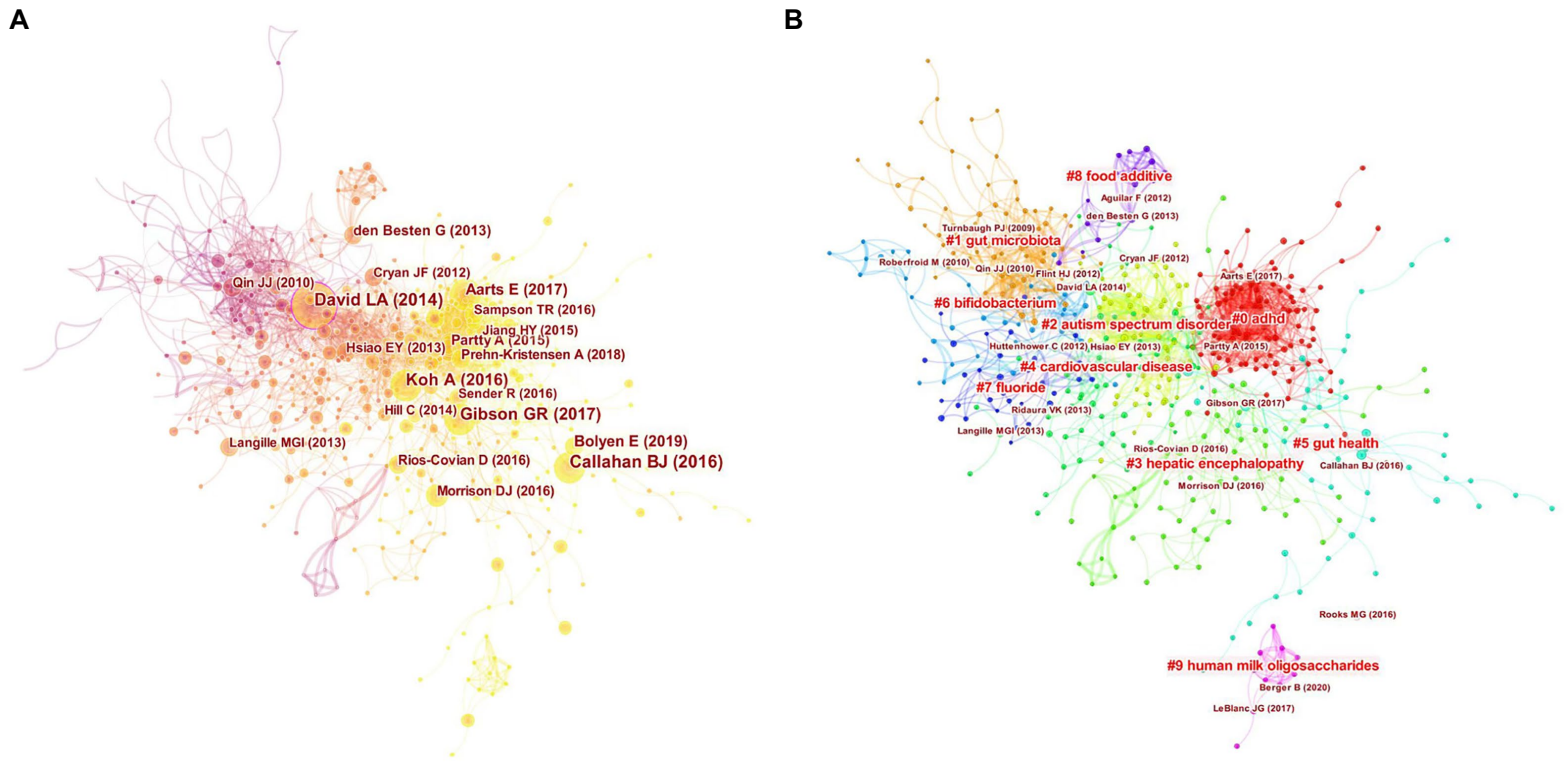
Analysis of references to gut microbiota in attention-deficit and hyperactivity disorder. **(A)** Network map of co-cited references. **(B)** Network map of co-cited clusters.

Table 4 shows the top 10 co-cited papers in the field of gut microbiota in ADHD and their citation counts, and central betweenness. Two of these 10 articles are from *PLoS One*, which may be one of the reasons for the highest number of co-citations to the journal above. It also shows that the journal has paid a lot of attention to the study of gut microbiota in ADHD. Most of the 10 articles are from the United States, where scholars have produced rich and widely recognized research results with high impact factors. The authors of two highly co-cited literatures (DAVID LA and Gibson GR) are also highly co-cited authors in this field. However, the number of co-citations of the top literatures with high co-citations is not large, indicating that this field still has room for development and tends to be mature. Figure 6A shows a visual network of co-citations, with darker to lighter colors reflecting the temporal variation of publications.

**Table 4:** Top 10 co-cited references.

Rank	Co-citation	Centrality	Author	Year	Title
1	56	0.18	David LA	2014	Diet rapidly and reproducibly alters the human gut microbiome
2	55	0.02	Callahan BJ	2016	DADA2: High-resolution sample inference from Illumina amplicon data
3	47	0.08	Koh A	2016	From Dietary Fiber to Host Physiology: Short-Chain Fatty Acids as Key Bacterial Metabolites
4	47	0.04	Gibson GR	2017	Expert consensus document: The International Scientific Association for Probiotics and Prebiotics (ISAPP) consensus statement on the definition and scope of prebiotics
5	38	0.09	Aarts E	2017	Gut microbiome in ADHD and its relation to neural reward anticipation
6	30	0.00	Bolyen E	2019	Reproducible, interactive, scalable and extensible microbiome data science using QIIME 2
7	23	0.05	Partty A	2015	A possible link between early probiotic intervention and the risk of neuropsychiatric disorders later in childhood: a randomized trial
8	23	0.06	den Besten G	2013	The role of short-chain fatty acids in the interplay between diet, gut microbiota, and host energy metabolism
9	21	0.00	Morrison DJ	2016	Formation of short chain fatty acids by the gut microbiota and their impact on human metabolism
10	13	0.02	Prehn-Kristensen A	2018	Reduced microbiome alpha diversity in young patients with ADHD

**Figure 7:**
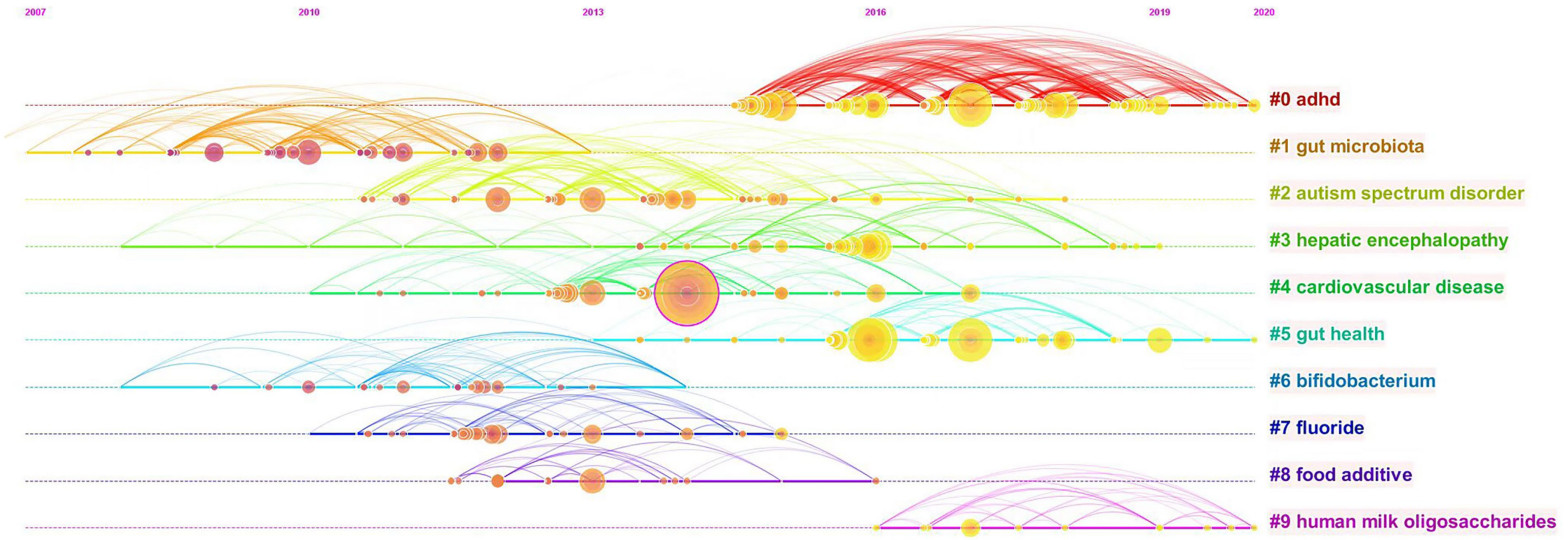
Timeline view of co-cited references related to gut microbiota in attention-deficit and hyperactivity disorder.

A simple co-citation analysis of the literature can only illustrate the connections between the literature and does not provide insight into the connections between the topics studied in the literature. As a result, we perform a cluster analysis on the co-citation network, and the literature is divided into different clusters, and those with similar research topics are divided into one category. We use CiteSpace to automatically name the different clusters, and the largest co-citation cluster is #0ADHD, indicating that this cluster has the largest number of co-cited literature (Table 5). This suggests that studies on the role of gut microbiota in improving ADHD symptoms have begun. The clusters are sequentially numbered from 0 to 9, and the number of included literature decreases in order. The silhouette values for all 10 clusters are close to 1, confirming a convincing delineation of the clusters. The year in the fourth column of the table is the average year of co-cited literature in the clusters, and the change in time also reflects the change in hotspots in the research field, corresponding to the Figure 7 timeline graph. Figure 6B reflects the top 10 clusters more visually. The different colors represent different clusters, and the major clusters are closely related.

**Table 5:** Top 10 largest clusters of co-cited references.

Cluster ID	Size	Silhouette	Year	Top terms
#0	110	0.917	2017	ADHD
#1	82	0.876	2010	Gut microbiota
#2	69	0.846	2013	Autism spectrum disorder
#3	69	0.917	2015	Hepatic encephalopathy
#4	64	0.858	2013	Cardiovascular disease
#5	48	0.905	2017	Gut health
#6	40	0.913	2011	Bifidobacterium
#7	36	0.913	2012	Fluoride
#8	17	0.952	2013	Food additive
#9	11	0.999	2018	Human milk oligosaccharides

Our timeline diagram using CiteSpace software helps us to get a clearer picture of the time span and development of the various research themes within the field of study (Figure 7). Combined with the timeline, cluster#1gut microbiota is the earlier research topic and is the foundation of the field of study. The horizontal axis also shows the amount of literature within each cluster, with each node representing a cited article and the node size representing how often that article has been cited. Cluster#0adhd has more nodes indicating that the field has developed a certain number of articles, while cluster#9Human milk oligosaccharides field is emerging. The solid line represents the duration of each topic, and the research frontiers in recent years have mainly focused on cluster#0adhd, cluster#5gut health, and cluster#9human milk oligosaccharides.

### 3.6. Keyword analysis

**Figure 8:**
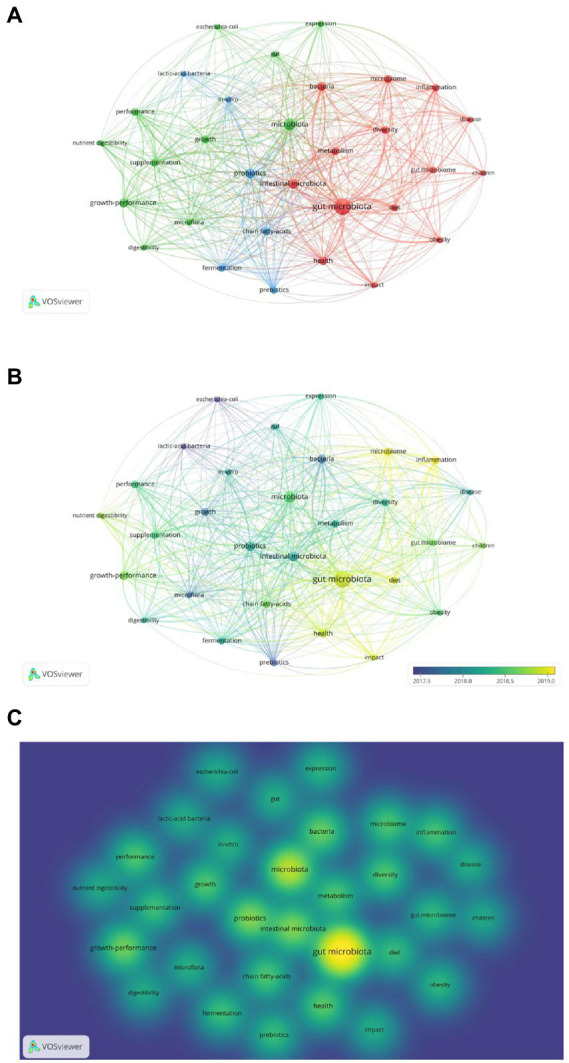
Analysis of keywords in publications related to gut microbiota in attention-deficit and hyperactivity disorder. **(A)** VOSviewer network visualization map of co-occurring keywords. **(B)** VOSviewer overlay visualization of co-occurring keywords by time. **(C)** VOSviewer density visualization map of co-occurring keywords.

Because keywords represent the focus and core content of an article, they are analyzed in order to facilitate the understanding of the research topic (Zhu et al., 2017). We used VOSviewer software and performed visual cluster analysis on the valid keywords to derive the research hotspots, as shown in Figure 8A. Different colors represent different clusters, which represent each hot topic in the field of gut microbiota in ADHD. The keywords in the figure are divided into three major clusters, which are related to gut microbiota, microbiome, and probiotics, respectively. Each node represents a keyword, and the larger the node indicates the more times the keyword appears in the 1975 literature, and the thickness of the connecting line represents how many times the two appear together in the same literature. The color in this Figure 8B from dark to light indicates that the keywords appear in increasing order of years. Since 2018, gut microbiota has become the biggest research hotspot in the field and diet is also beginning to be emphasized.

In density maps, the keywords of high co-occurrence appear with larger spots and the brighter the center of spot is, the higher betweenness centrality it has (Figure 8C). The density view can be used to get a quick look at important areas and the density of knowledge and research in a given area. The figure shows that there are more studies related to gut microbiota and microbiome.

**Figure 9:**
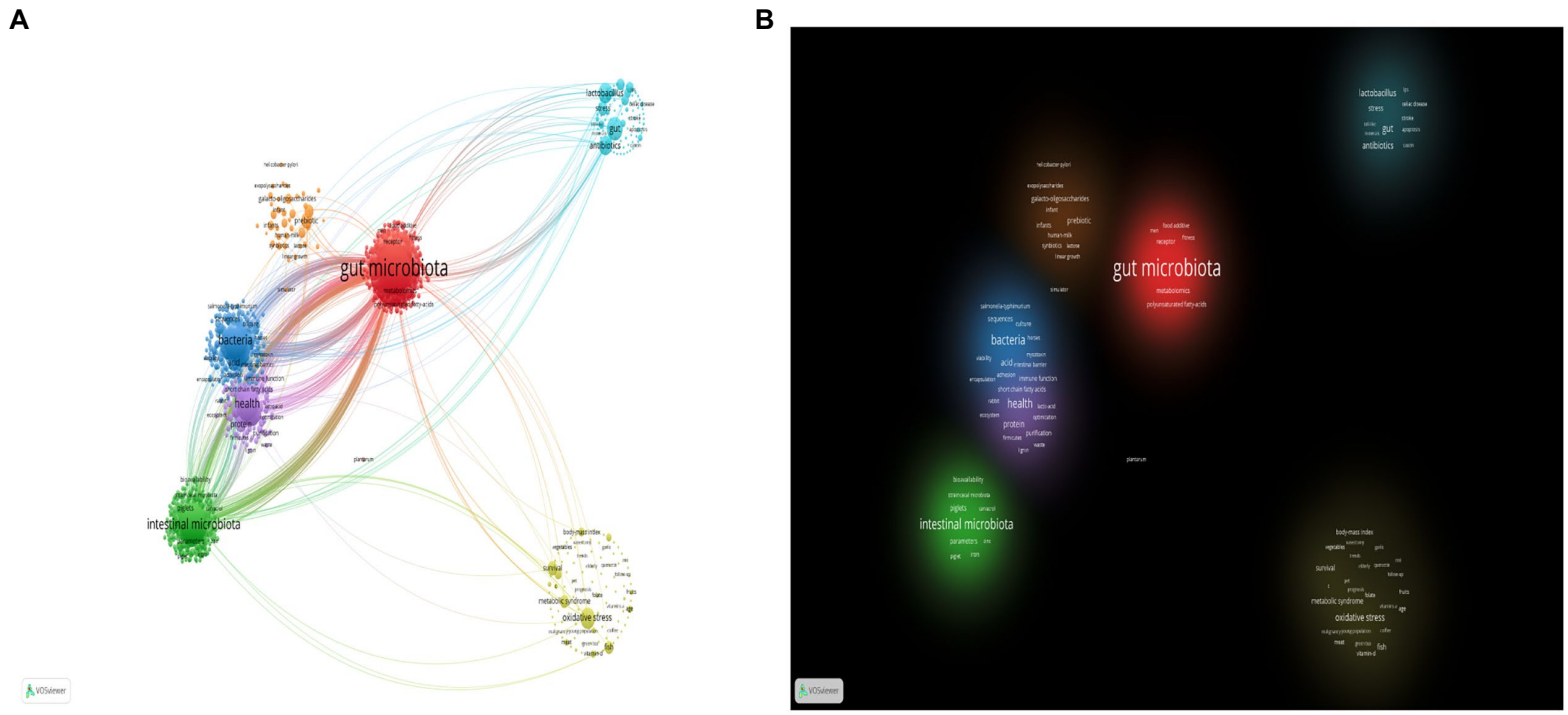
**(A)** VOSviewer and Pajek network visualization map of keyword clustering. **(B)** VOSviewer and Pajek density visualization map of keyword clustering.

At the same time, we used VOSviewer in conjunction with Pajek to produce a clearer and more esthetically pleasing cluster analysis map of keywords in the field (Figure 9). “Gut microbiota,” “bacteria,” “health,” “intestinal microbiota,” and “oxidative stress,” appeared more frequently.

### 3.7. Burst keywords analysis

**Figure 10:**
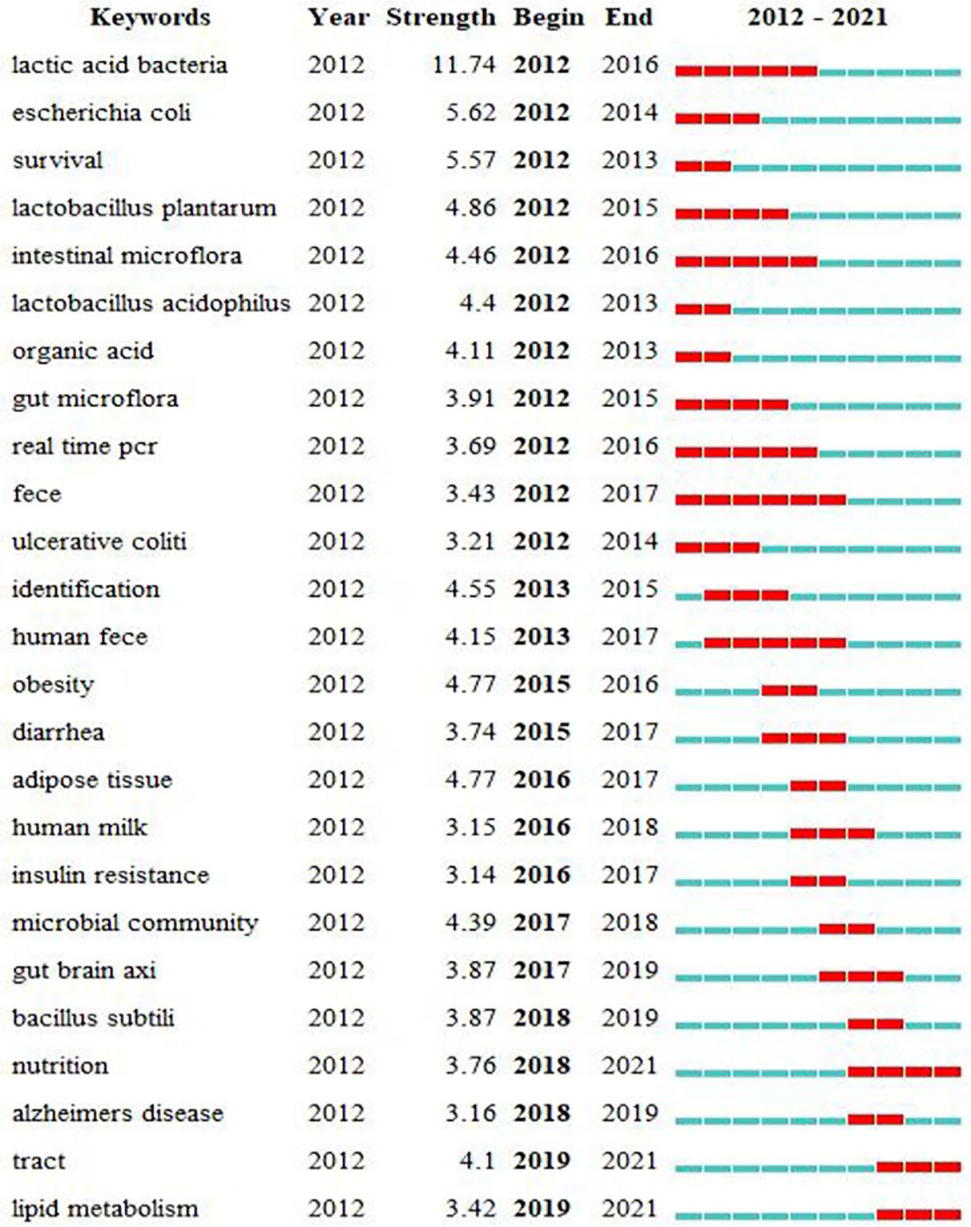
Burst keywords in articles related to gut microbiota in attention-deficit and hyperactivity disorder.

To get a clearer picture of the burgeoning research hotspots in the field, we used CiteSpace to generate keywords with high burst intensity, and the top ranking was lactic acid bacteria with a burst intensity of 11.74 for 5 years (Figure 10). This indicates that the use of probiotics to improve ADHD symptoms has long been a hot topic. The only keyword with a burst duration of more than 5 years was Feces (from 2012 to 2017). Fecal specimens are the most commonly used specimens in studies on topics related to gut microbiota (Scheperjans et al., 2015; Wan et al., 2022). During the period from 2012 to 2021, the keywords that emerged in this research area in the early period were lactic acid bacteria, escherichia coli, survival, lactobacillus plantarum, intestinal microbiota, etc., and the keywords that emerged in the middle term were obesity, diarrhea, adipose tissue, human milk, etc. The keywords that emerged in the late-term were bacillus subtili, nutrition, alzheimers disease, tract, lipid metabolism, etc. The table shows that nutrition, tract, lipid metabolism, and gut brain axis are emerging and active topics.

## 4. Discussion

### 4.1. Overall description

This study conducted a comprehensive and multi-angle metrological analysis of 1975 papers on gut microbiota in the field of ADHD retrieved from Web of Science Core Collection (WoSCC).

Through the trend fitting of the number of publications published from 2012 to 2021, it can be seen that the growth trend from 2015 to 2016 has turned (Figure 1A). Petra et al. (2015) explained the link between the gut-brain axis and the neuroimmune system, and that this interaction may influence the pathogenesis of inflammation-related diseases such as ADHD. This provides a theoretical basis for subsequent gut microbiota regulating ADHD. An article published in *Nature Microbiology* in 2016 describes the use of microbial culturomics, to culture the microbiota, increasing the number of cognitive gut microbiomes, thus making up for the lack of metagenomics (Lagier et al., 2016). This study broadens the horizon of understanding a variety of gut microbiota and lays the foundation for subsequent research on gut microbiota. The growth of the approximate index in recent years indicates that the field is maturing and still has great development potential.

In the analysis of countries and regions, the number of publications by country reflects, to some extent, the degree of contribution to the research field. Since the last decade, United States and China have made significant progress in this field of research (Figure 1B; Table 1). In our analysis of cooperation between countries, we find that international cooperation is not close. Most of the papers are still authored by scholars from a few countries, and it is important to strengthen cooperation and joint publications between countries to jointly promote the research progress in this field (Figure 2A). The citation/article ratio in China is the lowest among the top 10 high-yield countries. The late start of research in this field in China as a developing country may be one of the factors. Chinese scholars should pay more attention to the quality of research rather than the quantity (Figure 1C; Table 1). Also, three of the top 10 institutions in terms of number of publications are from China, which accounts for a relatively large share, indicating that some Chinese institutions are keen on the field (Table 1). This may be related to the increasing prevalence of ADHD in China in recent years. Moreover, Figure 3 suggests that the distribution of research papers on gut microbiota in ADHD among journals from 2012 to 2021 is approximately in accordance with Bradford’s law.

### 4.2. Analysis of author

From 2012 to 2021, there were 11,712 authors publishing in this field, with 0.17 articles *per capita*. By Price’s Law (G.F. Lotka, 1926), the minimum number of core authors in a field *m* = 0.749 × √(n _max_) ≈ 2.90 (n _max_ is the number of articles published by the most prolific author in the statistical year). The minimum number of publications for core authors was calculated to be 3. There were 436 core authors, accounting for 3.72% of all authors, and a total of 1898 publications, accounting for 96.1% of total publications. The *per capita* publication of core authors was 4.35, which was much higher than the *per capita* publication of all authors, which was 0.17. It can be seen that there is a stable group of authors in the field of gut microbiota in ADHD and they have strong research ability. In the co-analysis of the top 10 prolific authors and the top 10 co-cited authors, it was found that the number of co-citations of prolific authors was not high and that these authors focused on improving the impact of their research. Among the top 10 co-cited authors, DAVID LA (96 citations) and SCHLOSS PD (91 citations) were both from Harvard University in the United States, indicating that Harvard University ‘s research in this field has a strong influence (Table 3). Figure 5 shows a statistical mapping of the number of papers co-authored in different ways. We found that most of the national publications are generally domestic collaborations and fewer international collaborations, which also suggests that we should build international exchange and cooperation platforms and increase the frequency of international exchange so as to form stable and reliable cooperation relationships.

### 4.3. Analysis of co-cited references

We analyzed the most frequently co-cited papers in this field (Figure 6; Table 4), with the top two co-cited papers from the United States. The literature with the highest number of co-citations (56citiations) and the highest mediator centrality (0.18) is Diet rapidly and reproducibly alters the human gut microbiome published by David et al. (2014). In *Nature*, which details a series of experiments that demonstrated the ability of the gut microbiota to respond rapidly to dietary changes. The gut microbiota not only maintains normal physiological functions but also helps to fight against host infections. *Nature* has consistently provided cutting-edge and authoritative articles, and this research has had a strong impact. This study opens up ideas and provides theoretical support for subsequent dietary therapy to change the composition of the microbiota to regulate ADHD. The second is an article by Callahan BJ, who presents DADA2, an open-source R package that models and corrects Illumina-sequenced amplicon errors, which states that “DADA2 allows researchers to quickly and accurately identify microbial gene sequences and reconstruct them by amplification” (Callahan et al., 2016). The development of this new technology supports and provides the foundation for further studies of the microbiota. It has significance in understanding the pathophysiological mechanisms of different gut microbiota, searching for sensitive and specific molecular markers, providing reference for clinical disease treatment and improving the higher level of human understanding. The article published in cell by Ara Koh et al. analyzed in detail the synthesis, distribution, and mechanism of this energy and signaling molecule of SCFAs, suggesting that other intestinal flora metabolites may also have signaling roles (Koh et al., 2016). These studies in highly cited literature represent certain degree of research breakthroughs in different aspects, which have high academic value and can effectively promote the development of practical research. Later, from the time axis analysis of co-cited literature clustering (Figure 7), it is known that the focus of the study should be shifted from the early study of gut microbiota to the application of gut microbiota in disease treatment, which is undoubtedly a new breakthrough. Cluster # 0adhd, cluster # 5gut health, and cluster # 9human milk oligosaccharides are the main research directions currently detected. This may be because modulation of gut microbiota has been progressed in the treatment of other diseases, so the efficacy in neuropsychiatric disorders has also been studied. With the aim of treating neuropsychiatric disorders, correcting gut microbiota dysbiosis and achieving gut health, together with the presence of the gut brain axis, which affects brain development and behavior (Mathee et al., 2020). Research has also demonstrated that gut health plays a role in the immune system, respiratory system, central nervous system, and growth and development (Ronan et al., 2021). On the one hand, because the most abundant human milk oligosaccharides in breast milk is most closely associated with infant intestinal microbiota. On the other hand, the gastrointestinal tract is the best and most direct way to change the intestinal microbiota. Therefore, in recent years, scholars have intensively investigated the link between human milk oligosaccharides and gut microbiota, which have multiple physiological functions. In order to prevent a single disease or a combination of multiple diseases, such as ADHD, supplementation with human milk oligosaccharides can promote intestinal growth and development in infants as well as provide health benefits in adults (Šuligoj et al., 2020; Zhang S. et al., 2021). And we expect that these three research topics will continue to develop in the coming years.

### 4.4. Analysis of keywords

We performed clustering, time sequence, and density analysis on keywords in this field to identify different topics of research. In the early stage, it mainly focused on the basic research of “lactic acid bacteria, Escherichia coli, probiotics,” etc. In the middle stage, the role of short-chain fatty acids in the metabolites of the microbiota was noted. In the later stage, the effect of gut microbiota on disease and health status was studied (Figures 8, 9). Both the important role of gut microbiota in maintaining the health of the body and the proposed mechanism of the gut-brain axis has proven that gut microbiota can influence neuropsychiatric disorders, thus making this topic a hot topic. Researchers were able to classify bacteria using 16S ribosomal RNA amplicon sequencing (Szopinska-Tokov et al., 2020), which clarified the differences between the gut microbiota of ADHD patients and controls. Short-chain fatty acids, as microbial metabolites, are effective in promoting energy metabolism in mammals (den Besten et al., 2013). Metabolomics, a nascent histological technique, is able to identify metabolite signatures between host and microbiota (Morrison and Preston, 2016), which provides insight into how the gut microbiota functions. A microbiomics combined with metabolomics approach helps to gain insight into this research area and explore the mechanisms by which gut microbiota improves ADHD. In addition, the appearance of oxidative stress drew our attention as a possible mechanism for the pathogenesis of ADHD. Butyric acid, a short-chain fatty acid, causes mitochondrial dysfunction when its production is reduced, resulting in excessive ROS production (Mottawea et al., 2016), and when excessive reactive oxygen species are produced in the brain, oxidative stress can exacerbate neuronal damage leading to neuropsychiatric disorders such as ADHD, which is associated with oxidative stress (Corona, 2020). Early use of probiotic supplements has been documented to be useful in reducing the risk of developing ADHD (Ligezka et al., 2021). The diversity of gut microbiota depends on many factors, among which diet is a very important factor, so many scholars have studied it. Studies have shown that, Diets high in sugar and fat can increase the risk of ADHD, while healthy diets such as fresh fruits and vegetables can reduce the negative effects of ADHD (Del-Ponte et al., 2019), and in a study by Hiergeist et al. (2020) it was shown that antibiotics could reshape the composition of the gut microbiota and thus indirectly affect the development of ADHD.

### 4.5. Analysis of burst keywords

In the analysis of outbreak words (Figure 10), the emergence of “Alzheimer ‘s disease” indicates that scholars have noticed that the gut microbiota may affect the occurrence and development of central nervous disorder diseases, laying a foundation for the subsequent application of gut microbiota in the treatment of ADHD. We should pay attention to the words that are erupting and will continue to erupt, such as “nutrition, tract and lipid metabolism,” because these words are likely to indicate the direction of research for some time to come. The Academy of Nutrition and Dietetics recommends that adults and children should consume more plant-based foods and reduce high sugar and fat diets, which are beneficial in reducing the risk of chronic diseases. Furthermore, dietary fiber can regulate gut microbiota and promote microbiota balance (Dahl and Stewart, 2015). Currently, although diet is more important than supplement intake, nutritional supplements may be able to improve dietary efficacy (Howard, 2005). Besides, dietary Nutrient supplements have a good safety profile for patients and no serious adverse effects have been found, and can be used as an adjunct to the treatment of different mental disorders such as depression, schizophrenia, and ADHD (Firth et al., 2019). Emerging nutritional psychiatry opens new ideas for the prevention and treatment of mental disorders (Jacka, 2017). In conclusion, future research could focus on the feasibility of nutritional supplements for the treatment of ADHD.

What’s more, studies have shown that people with ADHD have a diet high in lipids, and that high total serum cholesterol and low-density lipoprotein are likely to contribute to the development of ADHD. Cholesterol circulating in the blood does not cross the blood–brain barrier and is not utilized by the central nervous system, and elucidating both the etiology and dietary control could help develop more effective treatments (Ugur et al., 2018). Lipids may not only be used to diagnose mental disorders, but may also be a cause of mental disorders (Grela et al., 2016). This is due to the fact that the brain is a highly lipid-dense organ and lipids are critical for brain and neurological health. Short-chain fatty acids, a product of diet and gut microbiota, also have varying effects on lipid metabolism (Morrison and Preston, 2016; Rios-Covian et al., 2016). Although studies on the mechanism of action of short-chain fatty acids are already available, it is necessary to continue to explore their effects on the basis of metabolomics. Vitamin D/Vitamin D receptor does have a protective effect on the nervous system of the brain and also regulates the gut microbiota, and VDR is indirectly linked to neurodevelopmental disorders through the gut brain axis (Ogbu et al., 2020). Vitamin D supplementation has been shown in trials to improve ADHD symptoms (Hemamy et al., 2021). It is also known that vitamin D is a lipid. In consequence, improving ADHD by regulating lipid metabolism is a possible future research direction, and abnormal serum lipids have potential as biochemical indicators of early ADHD. Researchers should further clarify whether lipid abnormalities in the brain are a cause of ADHD.

## 5. Limitation

First, although the Web of Science Core Collection sources have been more comprehensive and authoritative, the data in the database are always updated, and we cannot deny that some literature may be missed, which may result in an incomplete analysis of the data in the field. Secondly, when searching for literature, we only selected articles and reviews, and did not include other types of publications such as conference papers and online publications. Again, because the literature we analyzed was published from 2012 to 2021, the time course for understanding the progress of research on gut microbiota in ADHD may not be complete. Moreover, all of the literature in English was used for the metrological analysis in this paper, and no other languages were involved, which may have resulted in a biased analysis. Finally, although both CiteSpace and VOSviewer do provide powerful and flexible features to produce rich charts to visualize the knowledge graph and also help to understand a research field in a short time, the analysis of the literature can only be limited to an overview of the literature, which is still not enough for a detailed grasp of the research field compared to the traditional in-depth reading mode. The research field is still evolving and the software is being improved and updated. We should use knowledge mapping visualization to accurately grasp the key points of the research field and then conduct in-depth research and analysis in order to achieve groundbreaking research results.

## 6. Conclusion

This study provides the first comprehensive metrological analysis of the literature related to gut microbiota in ADHD from 2012 to 2021, using various visualization softwares to focus on publication volume characteristics, country/region collaboration, institutional journal authorship, author literature co-citation, and keyword outbursts to sort out the development of the field. During the decade, the number of publications in this field has steadily increased, and scholars from various countries have focused on this field, confirming the value of gut microbiota in treating ADHD. The gut microbiota can intervene early in the development of attention-deficit/hyperactivity disorder on the one hand and play a role in improving disease symptoms on the other. It can be said that the United States has a largely dominant role in this field, and it is necessary for scholars from various countries to develop more international cooperation, and China should focus on improving the quality and influence of articles. Among the detected outbreak words, “nutrition,” “lipid metabolism,” and “gut brain axis” are the frontier of current research and will continue to act. More importantly, researchers should strive to make the leap from microbiomics to metabolomics to fully unravel the association between gut microbiota and ADHD. Based on the research on the mechanism of gut microbiota in other diseases, there is reason to believe that the exploration of gut microbiota in ADHD must be increasingly mature. This work not only summarizes and analyzes the existing research in the field of gut microbiota in ADHD, but also indicates the direction of future research.

## Data availability statement

The original contributions presented in the study are included in the article/Supplementary material, further inquiries can be directed to the corresponding author.

## Author contributions

MZ and HC designed this study. YM drafted the original manuscript and revised it. BC is responsible for all the figures and tables in this article. All authors contributed to the article and approved the submitted version.

## Funding

This study was supported by a grant from the Natural Science Foundation of Hunan Province (2021JJ30995) and Science and Technology Innovation Program of Hunan Province (2020SK53617).

## Conflict of interest

The authors declare that the research was conducted in the absence of any commercial or financial relationships that could be construed as a potential conflict of interest.

## Publisher’s note

All claims expressed in this article are solely those of the authors and do not necessarily represent those of their affiliated organizations, or those of the publisher, the editors and the reviewers. Any product that may be evaluated in this article, or claim that may be made by its manufacturer, is not guaranteed or endorsed by the publisher.
