# Supplementary material for: A bibliometric analysis of studies on gut microbiota in attention-deficit and hyperactivity disorder from 2012 to 2021
Source: Front Microbiol. 2023 Mar 15;14:1055804. doi: 10.3389/fmicb.2023.1055804 (PMC10050751; doi:10.3389/fmicb.2023.1055804)

Supplementary Material

# Supplementary Table 1. The predicting the number of publications in 2022 related to gut microbiome in attention deficit and hyperactivity disorder.

| Year | Cumulative publications | Trend prediction | Lower confidence limit | higher confidence limit |
| --- | --- | --- | --- | --- |
| 2012 | 74 |  |  |  |
| 2013 | 157 |  |  |  |
| 2014 | 256 |  |  |  |
| 2015 | 361 |  |  |  |
| 2016 | 502 |  |  |  |
| 2017 | 695 |  |  |  |
| 2018 | 907 |  |  |  |
| 2019 | 1153 |  |  |  |
| 2020 | 1542 |  |  |  |
| 2021 | 1975 | 1975 | 1975 | 1975 |
| 2022 |  | 2402 | 2270 | 2534 |

# Supplementary Table 2. The Publications and Journals Count through Bradford's Law.

| Zone | Publications/Journal | Number of Journals | Number of Publications |
| --- | --- | --- | --- |
| First Zone | ≥11 | 30 | 662 |
| Second Zone | 2-11 | 138 | 663 |
| Third Zone | 1-2 | 549 | 650 |

# Supplementary Figure 1. The line chart that predicting the number of publications in 2022.


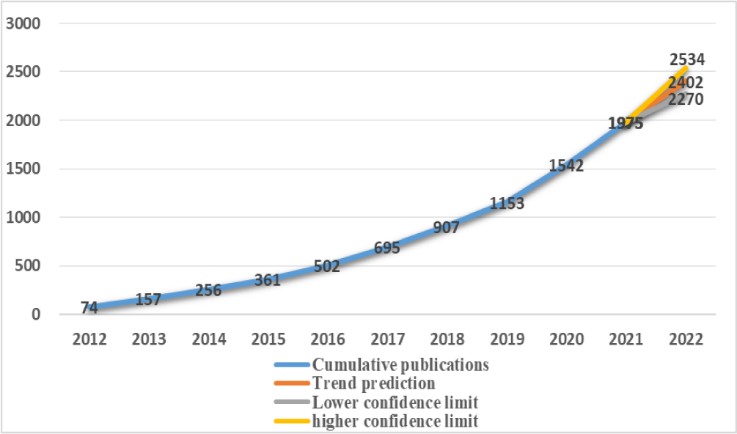

Supplement: Supplementary file 1 [file Table_1.DOCX]
